# Evaluating the effectiveness and risks of bread fortification programs in the middle eastern region: a comprehensive review

**DOI:** 10.3389/fpubh.2025.1530380

**Published:** 2025-03-11

**Authors:** Safa Abdul Majeed, Suad Said, Dana Ahmad Hassan, Faaiza Sadiq, Maryam Alhosani, Ayoub Al-Jawaldeh, Tahra El-Obeid, Reema Tayyem

**Affiliations:** ^1^Nutrition Sciences Department, College of Health Sciences, QU Health, Qatar University, Doha, Qatar; ^2^Regional Office for the Eastern Mediterranean (EMRO), World Health Organization (WHO), Cairo, Egypt

**Keywords:** bread, fortification, middle east, policy, vitamins and minerals, malnutrition

## Abstract

Fortification of staple foods such as bread has been implemented as a public health strategy to address nutrient deficiencies. Currently, 11 out of 22 Middle Eastern countries have mandatory wheat flour fortification, primarily with iron and folic acid, while others have voluntary initiatives. Despite progress in bread fortification efforts across the Middle East the long-term effectiveness and safety of these programs remain unclear. We assess the historical context, existing policies, and standards of fortification, focusing on public health outcomes, implementation challenges, and potential risks. A comprehensive search in PubMed, MEDLINE, Cochrane, and ProQuest databases, and relevant studies from the inception of the fortification efforts till 2024 were selected. Our search included Bread or flour fortification and their effectiveness and potential risks specific to Middle Eastern Regions. Findings from the literature indicate that fortification was effective in combating micronutrient deficiencies without causing the harmful effects of overload. However, ongoing research is needed to monitor potential risks, such as micronutrient overload. Most studies are concentrated in Egypt and Iran, with limited data from Saudi Arabia, Turkey, and Jordan, and a notable absence of research from other Middle Eastern countries. This highlights the need for further studies across a wider range of countries to provide a more comprehensive understanding of the regional impact and effectiveness of fortification policies. Addressing the challenges of inadequate coverage, compliance issues, and infrastructure limitations could provide a more comprehensive understanding of the region’s fortification policies and their effectiveness.

## 1 Introduction

Food fortification involves the addition of essential nutrients or bioactive components to edible products, particularly staple foods, to enhance their nutritional value and address nutrient deficiencies (1). Micronutrient deficiencies, often referred to as hidden hunger, remain a significant public health challenge globally, particularly in the Middle Eastern region. In the Middle East, iron deficiency is the most common problem that affects 30–70% of pregnant women and 10–50% of preschool children, while vitamin D deficiency impacts 46–83% of adolescents and adults. Additionally, 13.2 million preschool children have low serum retinol status, and 0.8 million children are affected by night blindness (2). Bread fortification has emerged as a widely adopted strategy to combat these deficiencies due to its accessibility and cultural significance as a dietary staple in many Middle Eastern countries (3).

Micronutrients play a major role in health outcomes and are crucial in the field of epigenetics. Notably, the vitamin B complex, which includes vitamin B6 (pyridoxine), vitamin B12 (cobalamin), folate (vitamin B9), choline, and methionine, is essential for metabolism, brain function, immune system support, and red blood cell formation (4). Additionally, these nutrients are critical for maintaining levels of S-adenosylmethionine (SAM), which is responsible for DNA methylation. This process is essential for regulating gene expression and maintaining cellular function. Altered DNA methylation has been linked to various diseases, including cancer, cardiovascular disease, autoimmune disorders, and neurological conditions (5). Adequate folate levels are also crucial during the rapid cell division that occurs in early embryonic development. Low concentrations of folic acid can impair DNA synthesis, leading to incomplete neural folds and can lead to defects such as spina bifida (incomplete closure of the spine) and anencephaly (absence of major portions of the brain and skull) (6). Other micronutrients, such as vitamin D, iron, iodine, and vitamin A, are equally vital. Vitamin D supports cardiovascular and bone health while regulating gene expression through its receptor (VDR) (7). Iron is essential for hemoglobin formation, iodine for thyroid function and brain development, and vitamin A for vision, immune function, and fetal development (8). Deficiencies of these micronutrients can cause altered metabolism, impaired growth and development, impaired cognitive function, decreased erythropoiesis, increased risk of infection, and decreased functional development finally leading to diseases such as Neural tube defects, Anemia, musculoskeletal disorders, dementia, loss of vision, and cardiometabolic diseases (9). While nutrient fortification programs aim to address deficiencies, their effectiveness, and potential risks need careful evaluation for long-term success and safety. Overconsumption off ortified nutrients can lead to health issues. For example, excessive folic acid intake may suppress immunity or mask vitamin B12 deficiency, leading to neurological disorders (10). A study in Iran found that iron-fortified flour reduced antioxidant capacity and increased oxidative stress without showing symptoms of iron overload (11). However, iron overload can elevate the risk of diabetes and heart problems in susceptible individuals. Additionally, high intake of certain vitamins, like beta-carotene, vitamin A, and vitamin E, may increase mortality, and too much calcium can lead to kidney stones. Additionally, the Middle East exhibits significant variation in food patterns and economic conditions. While some countries, like Turkey and Syria, have robust agricultural sectors, others rely heavily on imported food (12). Furthermore, micronutrient fortification is based on the Estimated Average Requirement (EAR), which meets the needs of 50% of the population. However, requirements can vary by age, gender, energy intake, and geographic location, so fortification may not be sufficient for everyone (13, 14). Currently, 11 out of 22 countries in this region have the mandatory fortification of wheat flour with essential nutrients mainly iron and folic acid. While some countries, such as Qatar and the UAE, have seen significant voluntary fortification efforts. The fortification standards was established ensuring compliance with WHO recommendations (15). The label regulation is set to increase emphasize consumer education about the benefits of fortified foods.

Despite the region’s progress in fortification efforts, disparities in outcomes and concerns regarding potential overconsumption, cost-effectiveness, and public acceptance are still unclear. These challenges highlight the need for a comprehensive review of existing literature to evaluate the effectiveness of bread fortification policies across the region. Therefore, this study aims to assess the public health outcomes, implementation challenges, and potential risks associated with bread fortification programs in the Middle East.

## 2 Methods

A comprehensive literature search was conducted in PubMed, MEDLINE, Cochrane, and ProQuest databases, as well as studies published in Arabic and English, focusing on Middle Eastern countries, from 1990 (beginning of the fortification era) to 2024. The selected databases—PubMed, MEDLINE, Cochrane, and ProQuest—are widely recognized for their extensive coverage of high-quality peer-reviewed articles across various disciplines, including nutrition and public health. The following terms were used in the search strategy: “Bread fortification OR flour fortification OR micronutrient enrichment OR micronutrient encapsulation OR food fortification” and effectiveness and public health outcomes. In addition, relevant resources from the World Health Organization (WHO) and organizational websites were also consulted. To ensure a comprehensive topic analysis each author independently performed the literature search, and Table 1 provides a summary of the search strategy.

**Table 1:** Summary of search approach.

List	Details
Search date	From March to May 2024.
Databases	PubMed, MEDLINE, Cochrane, ProQuest, and various databases related to public health, nutrition, and food fortification studies published in Arabic and English. World Health Organization (WHO) and organizational websites: Food fortification initiative; https://www.ffinetwork.org/ Global fortification Data; https://fortificationdata.org/
Search terms	“Bread fortification OR (flour fortification)) OR (micronutrient enrichment)) OR (micronutrient encapsulation)) OR (food fortification)))) AND (effectiveness)) OR (impact)) OR (risk)) OR (public health outcomes)) OR (adverse effect)) OR (policy)))) AND (middle east)) OR (gulf countries)) OR (arab world)) OR (east mediterranean region)) OR (qatar)) OR (bahrain)) OR (egypt)) OR (iran)) OR (iraq)) OR (syria)) OR (jordan)) OR (kuwait)) OR (lebanon)) OR (oman)) OR (palestine)) OR (saudi arabia)) OR (yemen)) OR (united arab emirates)) AND (iron)) OR (vitamin D)) OR (zinc)) OR (folate)) OR (vitamin B complex)) OR (vitaminA)
Time frame	From inception to 2024, to ensure up-to-date information.
Inclusion/exclusion criteria	All study designs except for narrative/literature review relevant to the topic of interest.
Collection procedure	The literature search was conducted independently by all authors

## 3 Results

### 3.1 Early attempts in bread fortification

Earlier, bread fortification was initiated during the early civilization by adding ingredients such as milk, honey, and various herbs and spices to enhance its flavor and nutritional value. Although these efforts were not based on scientific knowledge, they laid the foundation for a later systematic approach to bread fortification. Nowadays, the latest fortification technology involves either fortifying the flour or applying microencapsulation to include bioactive substances such as vitamins and minerals in bread without negatively impacting its properties (16).

Initially Bread Fortification program was introduced in Oman in 1996, and it is then followed by other countries. Fortifying the flour with iron and folic acid was introduced primarily to address increased neural tube defects (NTDs) and iron deficiency anemia in this region.^1^ In Egypt, the prevalence of NTDs during pregnancy was 4.5 per 1,000. At Giza hospital, the incidence rate of birth defects among 3,000 newborns was 3.17% (17). Anemia was also a major public health problem in this region. During the EDHS 2005 survey the prevalence of anemia increased from 37 to 52% among Egyptian children between 12 and 36 months of age while in married women aged between 15 and 49, it increased from 28% in 2000 to 39% in 2005 (18). Zinc deficiency was also more prevalent during this s period with 19.3% of pre-school children in Iran (19). All these nutritional problems in these countries in the early 20th century led to mandatory wheat flour fortification with iron and folic acid covering 82% of wheat flour mills and mandates the fortification as part of legislation from 2005 (15, 20) in staple foods such as bread, which was widely used in households.

The focus on reducing NTDs through folic acid fortification is particularly relevant given that these defects often result in severe disabilities or mortality shortly after birth. NTDs a severe congenital malformation resulting from the failure of the neural tube, the precursor of central nervous system, to close properly during early embryonic development due to deficiency in Foli acid leading to spina bifida (incomplete closure of the spine) and anencephaly (absence of major portions of the brain and skull).

In 2008, the Government of Egypt launched a 5-year national program of fortification of iron and folic acid in wheat flour used to produce subsidized Baladi bread, the staple food consumed by many low-income groups in Egypt. This policy program was triggered by the 2005 Egyptian Demographic and Health Survey (EDHS) results. As a result, the Ministry of Supply and Internal Trade (MOSIT) mandated fortification in all private and public sector mills (20).

In 1994, Iran initiated a national nutrition strategy which received support from international organizations like WHO and UNICEF. By 1998, a refined strategy was developed with support from the Micronutrient Initiative (MI) and the World Bank, leading to a pilot trial in Isfahan that showed improved iron status. Subsequent workshops and collaboration with flour millers helped expand the program nationwide by 2004. Strong political commitment, multi-sectoral collaboration, and social marketing were key to the program’s success. In 2005, Iran passed a law requiring wheat flour fortification, which was implemented nationwide by 2007 (21). In addition, earlier in 1973, bread fortification of lysine was done in Iranian bread as a school lunch for pre-school children (22). Both countries’ policy included regulatory measures to ensure the consistency and quality of fortification, and labeling requirements were introduced to inform consumers about the nutritional content of the bread. These measures were based on WHO recommendations.

### 3.2 Current bread fortification policies in the middle east

#### 3.2.1 An overview of existing fortification programs in different countries

In the Middle East, policies regarding bread fortification vary across countries, ranging from voluntary initiatives to mandatory programs, each tailored to address specific nutritional deficiencies and improve public health outcomes (23–25). Qatar, for example, has voluntary fortification initiatives in place since 2015, achieving 90% coverage through industrial mills (23, 26, 27). Similarly, Saudi Arabia has fortified all industrially milled flour with essential nutrients since 2015, achieving 100% coverage and the potential for significant health impact (23, 24).

Also, in Kuwait, the voluntary fortification program was legislated in 2015 mandates the fortification of wheat flour with many nutrients, and it achieves full coverage through a single industrial mill (23, 26). Reflecting a program that is well-established and closely monitored. In the United Arab Emirates (UAE), a voluntary program has resulted in 90% of industrially milled flour being fortified with essential nutrients (23, 24, 26). In 2000, Iraq introduced voluntary flour legislation under the governance of Iraqi Standard Specification (28), later receiving UNICEF support in 2006 aimed at combating nutritional deficiencies (26), despite legislative efforts and the presence of 300 industrial mills processing all flour, none of the industrially milled flour undergoes fortification, resulting in a 0% fortification rate in this sector (23) In contrast to these voluntary approaches, Oman stands out with its longstanding mandatory fortification program initiated in 1996 (27) and implemented the wheat flour legislation in 1997. In 2010, Oman integrated iron and folic acid into all domestically produced and imported wheat flour, while in 2022, new regulations expanded fortification to include various flour types and additional nutrients like vitamin D3 and vitamin B12 (29). Comprehensive legislation and integration into the health financing system ensure broad coverage and sustainability. Almost all (89% according to Food Fortification Initiative; FFI) wheat flour is industrially processed and available for fortification (23, 24). Bahrain similarly enforced mandatory wheat fortification in 2002 (24), targeting anemia and neural tube defects, yet faces challenges such as low coverage among small-scale mills (26, 27). However, 90% of the industrial flour is fortified in Bahrain according to FFI (23, 24). Meanwhile, Yemen has required 100% of industrially milled wheat flour to be mandatorily fortified with iron since 2001, following WHO recommendations to address nutrient deficiencies in vulnerable populations (23, 26). In Iran, wheat flour fortification has been mandatory since 2006. This covers all domestically produced wheat flour types, including all domestically produced wheat flour types, with a reported coverage of 335 grams per capita per day (24, 27). Moreover, A national flour program for fortifying has been initiated in Jordan since April 2006 (26), while the mandatory legislation was implemented in 2008 (24), achieving 93% coverage and significantly reducing iron deficiency and anemia, serving as a regional model emphasizing coordinated efforts and sustainable funding (23, 26). Since 2005, Palestine has mandated fortification (30), despite challenges that Palestine faces, such as insufficient data on coverage and intake (26).

In Syria and Lebanon, the absence of comprehensive fortification programs, particularly in wheat flour, poses a public health concern, placing the population, especially vulnerable groups, at risk of various health issues (24, 27). In Syria, a wheat flour fortification program initiated in 2003 showed promising results by 2009 in reducing iron-deficiency anemia, but it was suspended in 2011 due to the crisis, leading to a setback in expertise and momentum (31). Meanwhile, Lebanon’s lack of fortification legislation for flour is due to disagreements that led to discussions on recommending fortification, addressing barriers, and exploring fluoridation. Similarly, Türkiye lacks a national program to fortify wheat flour. All flour produced in the country is from industrial mills without fortification (24). However, there are indications of a possible mandatory fortification program in the future (27).

#### 3.2.2 Specifications of fortification standards

In the Middle East, wheat flour fortification standards vary across different countries, yet they share commonalities in addressing nutritional deficiencies and improving public health outcomes (32). These standards are established based on international guidelines and recommendations, ensuring safety and effectiveness in fortification practices (26, 27). Wheat flour fortification typically includes essential nutrients such as iron, folic acid, and B-complex vitamins tailored to meet population needs (2, 33). Fortification standards for wheat flour vary across countries, each with its own set of regulations and compliance measures.

Wheat flour fortification standards in Qatar, Saudi Arabia, the UAE, and Kuwait mirror each other, following the GCC (Gulf Cooperation Council) Standardization Organization (GSO) standards (24), this standard aims to address nutritional deficiencies and requires food products for dietary use to have nutrition fact panels (23, 27, 34). Specifically, these countries fortify wheat flour with calcium (2,115 mg/kg), folic acid (1.75 mg/kg), iron (30 mg/kg), niacin (52.91 mg/kg), riboflavin (3.96 mg/kg), thiamine (6.38 mg/kg), and vitamin D (0.0137 mg/kg) to address common deficiencies and enhance public health (24). These standards are in line with international recommendations and serve as vital regulatory frameworks ensuring consistency and effectiveness across the region (27). While in Iraq, the voluntary fortification of wheat has standards for wheat, including adding folic acid at a level of 2.1 mg/kg and adding ferrous sulfate at a level of 45 mg/kg (24). However, local flour lacks iron fortification, while imported flour meets Iraqi standards (27). Iron and folic acid in flour help reduce anemia cases and have health benefits (16).

The mandatory fortification standard in Oman includes folic acid 1.5 mg/kg and 60 to 120 mg/kg iron (23, 24). Similarly, Bahrain’s mandatory fortified wheat flour with 1.5 mg/kg of folic acid and 60 mg/kg of iron aims to reduce anemia and neural tube defect rates (24, 26). In addition, Yemen enforces mandatory fortification of all industrial wheat flour, incorporating iron and folic acid to combat widespread nutrient deficiencies (26), particularly among vulnerable populations like children and pregnant women (27). The government’s resolution, issued by the Prime Minister in 2001, made it mandatory for wheat flour to be fortified (14). The fortification standards include folate (1.5 mg/kg) and iron (60 mg/kg), with all wheat flour being fortified (14). In Jordan, the fortification standards include essential nutrients such as 34 mg/kg of iron in the form of ferrous sulfate, 14.15 mg/kg of calcium, 1.52 mg/kg of folic acid, 20 mg/kg of zinc, 0.008 mg/kg of vitamin B12, 35 mg/kg of niacin, 3.6 mg/kg of riboflavin, 2.89 mg/kg thiamine, and 1.5 mg/kg of vitamin A (24, 33). In Palestine, mandatory fortification of wheat began in September 2005 (30); while fortifying the wheat flour with 3.6 mg/kg of vitamin B6, Palestine shares other nutrients added to the wheat flour in the same content as Jordan (24).

Lebanon currently lacks both mandatory and voluntary wheat flour fortification programs (26). However, the daily per capita wheat flour consumption of 233 grams presents a significant opportunity for potential fortification interventions (24). Syria presents a contrasting scenario, where fortification data is limited, highlighting the need for improved implementation (24). Despite an industrial processing rate of 82%, only 5% of wheat flour undergoes fortification, indicating substantial room for enhancement (24, 27). Türkiye currently also lacks standards for wheat fortification due to the absence of a national fortification program for wheat flour (24, 27).

In a broader context, fortification standards in the Middle East are developed based on population needs and international guidelines to ensure safety and effectiveness. These standards encompass recommended amounts and permissible ranges of added nutrients, considering factors such as bioavailability and organoleptic characteristics to optimize health benefits while minimizing the risk of toxicity. Furthermore, labeling requirements, claims, and advertising guidelines are integral components of these standards, serving to inform consumers about the fortified products and promote transparency in the marketplace. The fortification standards of different countries are summarized in Table 2.

**Table 2:** Comparison of micronutrient fortification standards for bread across middle eastern countries.

Country	Iron (mg/kg)	Folic Acid (mg/kg)	Vitamin D (mg/kg)	Zinc (mg/kg)	Vitamin A (mg/kg)	Calcium (mg/kg)	Niacin (mg/kg)	Riboflavin (mg/kg)	Thiamine (mg/kg)	Vitamin B6 (mg/kg)	Vitamin B12 (mg/kg)
Qatar	30	1.75	0.0137	N/A	N/A	2,115	52.91	3.96	6.38	N/A	N/A
Saudi Arabia	30	1.75	0.0137	N/A	N/A	2,115	52.91	3.96	6.38	N/A	N/A
UAE	30	1.75	0.0137	N/A	N/A	2,115	52.91	3.96	6.38	N/A	N/A
Kuwait	30	1.75	0.0137	N/A	N/A	2,115	52.91	3.96	6.38	N/A	N/A
Oman	60–120	1.5	N/A	N/A	N/A	N/A	N/A	N/A	N/A	N/A	0.008
Bahrain	60	1.5	N/A	N/A	N/A	N/A	N/A	N/A	N/A	N/A	N/A
Yemen	60	1.5	N/A	N/A	N/A	N/A	N/A	N/A	N/A	N/A	N/A
Jordan	34	1.52	0.0145	20	1.5	14.15	35.00	3.60	2.89	3.62	0.00763
Palestine	34	1.52	0.023	20	1.5	15	35.00	3.60	2.89	3.6	0.004
Iraq	45	2.1	N/A	N/A	N/A	N/A	N/A	N/A	N/A	N/A	N/A

#### 3.2.3 Compliance and enforcement mechanisms

Compliance and enforcement mechanisms for wheat flour fortification policies in the Middle East are crucial for addressing nutritional deficiencies and improving public health outcomes. Regulatory oversight varies across countries, but common mechanisms include government inspections (32, 35). Additionally, in various Middle Eastern countries, regulatory oversight is typically overseen by government agencies or ministries responsible for public health, food safety, or standardization (26, 27, 30, 35). For instance, in Qatar, the Ministry of Public Health (MOPH) oversees compliance and enforcement of wheat fortification regulations (36). Similarly, in Jordan, the Ministry of Health is responsible for the implementation of fortification programs, with the Jordan Food and Drug Administration (JFDA) monitoring compliance with regulations (2, 35). These governmental bodies often conduct inspections and technical audits at flourmills to ensure compliance with fortification standards (26, 27, 35). Manufacturers must follow Good Manufacturing Practices (GMP), Hazard Analysis and Critical Control Points (HACCP), or International Organization for Standardization (ISO) guidelines to fortify products and ensure safety (26).

Kuwait achieves full compliance through rigorous monitoring and quality control processes despite the absence of mandatory fortification (27). In contrast, some countries have specific legislation mandating fortification, such as Bahrain, Iran, Jordan, Oman, Palestine, and Yemen, where fortification is mandatory by law (24). Integrating it into the health financing system and enforcing compliance through government regulations and monitoring efforts (27). In addition, in Bahrain, the Public Health Directorate’s food inspection section conducts regular monitoring and enforcement by visiting mills every 2 months to ensure a high compliance rate, with compliance rates typically exceeding 90% (26). Noncompliance penalties may vary across countries and include fines, operations closure, or other regulatory actions (26). In Jordan, for example, bakeries found to be operating without a license, not following process hygiene regulations, or using non-fortified flour may face fines or closure of their operations (2).

Despite these efforts, challenges persist in ensuring widespread compliance. In Iraq, compliance with fortification standards is reported to be at 0%, indicating a lack of fortification in locally produced flour (28). Moreover, compliance with fortification programs in Palestine faces significant challenges, including low adherence rates despite mandatory legislation and ongoing monitoring efforts (26), despite the reported 100% coverage (24). Similarly, in Syria, compliance with fortification standards is low, with only 5% of flour estimated to be fortified. Still, given that 82% of wheat flour is industrially processed, there is significant potential for fortification to improve national health outcomes (24, 26, 27). In Lebanon, there is no fortification program, which requires policy reforms and better monitoring. However, difficulties such as standardizing, coordinating, and testing for iron in wheat flour make it challenging and costly (24, 26) Although there is currently no wheat legislation in Türkiye (24), the Turkish Flour Industrialists’ Federation (TFIF) is promoting the adoption of fortification practices by fortifying wheat flour (37).

### 3.3 Impact of bread fortification policies on health and its effectiveness

**Figure 1:**
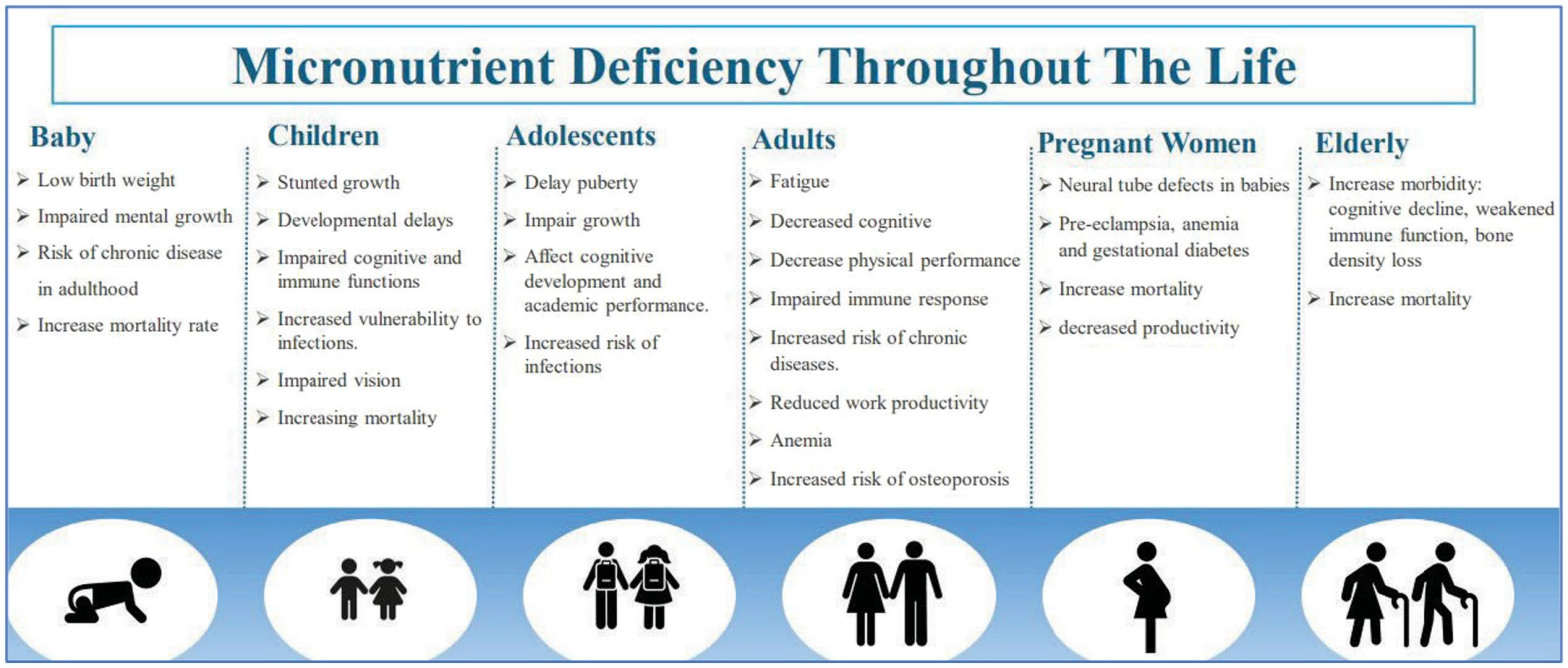
Micronutrient deficiency throughout life. Data adapted from Kiani et al. (38).

The World Health Organization (WHO) identifies malnutrition as an umbrella term that covers both Undernutrition and over-nutrition. Undernutrition is a specific concern where the body lacks essential building blocks (36). This includes protein and micronutrients like Iron, vitamin A, Iodine, folate, and Zinc. Micronutrient deficiencies has demonstrably negative consequences for human health and is mainly seen among pregnant women and children below 5 years of age. These include intellectual impairment, perinatal complications and poor growth. As in pregnancy, neural tube defects which can result in preterm delivery and fetal growth retardation while in children it leads to impaired cognitive development (38). Furthermore, micronutrients such as vitamins and minerals required in small amounts are crucial for producing enzymes, hormones, and other substances vital for proper growth and development (39). The absence of these micronutrients can escalate morbidity and mortality (38) (Figure 1). Poverty, which limits access to a variety of foods, or simply a monotonous diet can all contribute to Undernutrition. Fortified bread serves as an effective intervention to combat undernutrition, especially in vulnerable groups like children and pregnant women (40).

Therefore, Governments in the region have implemented fortification programs for wheat flour used in bread baking, as bread is a staple food consumed in enormous quantities to improve the overall vitamin and mineral status of populations.

#### 3.3.1 Bread fortification with iron

Iron deficiency is a major concern in this region contributing to a global health challenge known as iron deficiency anemia which mainly effects children, pregnant women, and individuals in low-income countries. The prevalence of iron deficiency varies across countries in the Middle East. According to 2019 survey the prevalence of anemia in Egypt is 28.3% among women of reproductive age (15–49 y); 32.3% among children under five; 26.0% among pregnant women; and 28.4% among non-pregnant women (41). Current prevalence of each country is mentioned in the supplementary material.

Iron plays a crucial role in immune function and red blood cell production therefore its deficiency compromises the body’s ability to combat infections and transport oxygen to tissues leading to fatigue, weakness, and impaired cognitive development in children. Targeted dietary interventions are critical in addressing this issue and mitigating its impact (42, 43). Fortifying bread with Iron is a promising strategy to increase overall iron intake and improve iron status.

A study in Iran evaluating the impact of iron-fortified bread found that while it improved serum ferritin levels, it did not significantly reduce anemia rates. Similarly, ‘Before-and-after’ studies on the flour fortification program with iron in Bushehr and Golestan provinces, involving 600 and 652 women respectively, showed a beneficial effect on ferritin levels but did not significantly alter the prevalence of iron-deficiency anemia in either province (44, 45). It can be justified through various reasons. Firstly, flour fortification of Iron only addresses anemia caused by iron deficiency, but Anemia can also be caused by other micronutrient deficiencies. Secondly, increased inflammation due to infection and obesity can reduce iron absorption. Thirdly, Iron fortification compounds have varying absorption capacity some foods undergo undesirable color or flavor changes when iron is added, and many iron-fortified foods contain inhibitors of iron absorption (46). Specific iron compounds, such as sodium iron (III) ethylenediaminetetraacetate, offer advantages as they enhance absorption even from high phytate foods, which can otherwise hinder iron uptake (47). However, acceptable daily intake (ADI) for humans is 1.9 mg per kg of body weight per day (48). Therefore, improving sanitary practices and infection control, ensuring clean drinking water, and reducing obesity rates will likely have a more significant impact on improving iron levels and reducing anemia in these regions.

The success of Jordan’s iron-fortified bread program is evident in the reduction of both iron deficiency and iron deficiency anemia (IDA). According to the Jordan National Micronutrient Survey (JNMNS) 2019 out of total participants only 28.6 and 10.7% had iron deficiency and iron deficiency anemia respectively, which is less than previous estimate of 2002–2010 survey. In addition, the survey also found that Children aged 12–59 months obtained about two-thirds of their recommended iron intake from fortified bread (49, 50) Moreover, a recent study that looked at the side effects of fortification of Iron in commonly used types of Iranian flour and bread (Barbary, Lavash, and Tafton) in Isfahan, Iran, found that for adults, the risk of getting too much Iron from eating fortified bread was low. However, a study in Semnan, Iran, found that flour fortification with iron led to reduced antioxidant capacity and increased oxidative stress biomarkers in non-anemic healthy men, although no symptoms of iron overload were observed (11). For children, who are more sensitive to Iron, the risk was higher and could be harmful. The researchers suggested more studies that should be done to make sure that the iron levels in fortified bread are safe, especially for children (51). In addition, Mohammadi et al. explored iron levels in wheat flour, a critical dietary source in iron-deficient Iran. Iron content in various Tehran wheat flours fell short of the Iranian health ministry’s minimum requirement of 40 ppm despite fortification with 30-ppm ferrous sulfate. Furthermore, the study revealed variations in iron content between different flour types (52). These findings emphasize the need for stricter monitoring and potentially revised fortification strategies by ensuring adequate iron compound selection, proper fortification levels, and consistent monitoring across all flour varieties, countries as Iran can harness the full potential of flour fortification to combat iron deficiency and its associated health problems.

Some experts worried that low-income individuals might consume too much Iron from fortified bread, leading to iron overload and potential harm to individuals with certain medical conditions. However, research and survey data showed that fortification was effective in combating iron deficiency without causing harm, although ongoing research was monitoring for potential iron overload (53).

#### 3.3.2 Bread fortification with zinc

Zinc is a crucial mineral for maintaining immunity, growth, and development. It is important to maintain a consistent dietary intake of zinc as our bodies do not store zinc efficiently. Zinc deficiency can lead to stunted growth, weakened immunity, and slow wound healing, especially among children in the Middle East, where micronutrient deficiencies are prevalent. Palestine has the highest prevalence of zinc deficiency among preschool-age children at 55.6%, with female adolescents accounting for 70% and male adolescents at 58.6%, as well as pregnant women at 71.1%. Meanwhile, Somalia has the lowest prevalence of zinc deficiency among preschool-age children at 5%. However, Afghanistan reported a higher zinc deficiency prevalence than Jordan, with rates of 23.4 and 22.1%, respectively, among women of reproductive age. The zinc deficiency rate among pregnant women in Pakistan and Iran is 37.2 and 28%, respectively (8).

Food fortification, which involves adding zinc to processed food, is a cost-effective solution to this problem (54–56). Zinc fortification of bread has been an effective strategy to address zinc deficiency. In Iran, a randomized clinical trial was done to assess the effect of consuming zinc-fortified bread on serum zinc and iron status of zinc-deficient women. Results showed that the group consuming high-zinc bread had significantly greater zinc and iron absorption compared to the low-zinc group. The study concluded that fortifying flour with 50–100 ppm zinc was effective in improving zinc intake and absorption in zinc-deficient individuals. Additionally, consuming zinc-fortified bread improved iron absorption (57). Estimates show that the consumption of Iranian flat breads can provide around 3.5 mg of zinc per person per day, making breads a reliable source of dietary zinc (58). Zinc fortified bread was also introduced in Türkiye. In a study, school children with low serum zinc concentrations received zinc-fortified bread providing 2 mg/kg/day for 90 days, whereas the other children received the same quality bread with no zinc fortification (control group). Results showed that by the end of the period, the zinc-supplemented group had significantly higher serum and leukocyte zinc concentrations indicating that the bioavailability of zinc in the bread is satisfactory, and the use of zinc-fortified bread is an accessible method to eliminate zinc deficiency and to prevent further occurrence (59).

#### 3.3.3 Bread fortification with vitamin D

Vitamin D is crucial for bone and muscle health, functioning more like a hormone by regulating calcium and phosphate levels in the body. Deficiency can result in bone osteomalacia in adults and rickets in children. Recent studies have associated low vitamin D levels with an increased risk of chronic diseases like cancer, high blood pressure, and autoimmune disorders (60, 61). In the Middle East, despite the abundant year-long sunshine, there is a high prevalence of vitamin D deficiency and inadequacy. Traditional clothing that covers most of the body and the lack of foods that are either rich in vitamin D or fortified with it can contribute. When reviewing the available data on vitamin D deficiency in Middle Eastern countries, it was challenging to make comparisons due to the use of diverse cut-off points to define deficiency and the varied assays used to measure it. In different countries, the levels of vitamin D deficiency and insufficiency vary widely among various age groups. In Jordan, both preschool and school-age children have high deficiency rates (27.7 and 44.2%, respectively) and insufficiency rates (44.2 and 43.3%, respectively). Afghanistan follows with a significant deficiency among preschool children (16.8%) and high insufficiency rates across all age groups (64.2% in preschool children and 64.7% in non-pregnant women). Palestine reports lower deficiency rates (6% in preschool children) but higher insufficiency rates (54.7% in preschool children and 99.3% in pregnant women). Oman shows a moderate prevalence of insufficiency (53.8% in preschool children and 41.5% in non-pregnant women). Morocco presents lower insufficiency rates compared to other countries (23.4% in preschool children and 47.5% in non-pregnant women). Iraq records severe deficiency among non-pregnant women (74.5%), while Pakistan reports high insufficiency rates (54% in preschool children and 85.3% in pregnant women). Iran’s data primarily focuses on pregnant women and shows a combined rate of insufficiency and deficiency of 81.2% (8). To address these, researchers explored vitamin D-fortified bread.

A randomized double-blind trial was done to assess the bioavailability of vitamin D from fortified Iranian bread and its effects on health. 90 healthy participants aged 20 to 60 years were divided into 3 groups: The fortified bread group (FP), the Placebo group (vitamin D supplement-SP), and the Plain bread group (CP) over 8 weeks. Results showed that changes in serum 25-hydroxyvitamin D concentrations were significant in the FP and SP groups, increasing by 39.0 ± 22.6 nmol/L and 28.9 ± 31.2 nmol/L, respectively, compared to a decrease of 9.2 ± 12.3 nmol/L in the CP group. The study concluded that vitamin D-fortified bread could effectively increase circulating 25-hydroxyvitamin D levels in the population to an adequate level, and it also showed potential benefits for other health aspects such as PTH levels, visceral fat, and lipid profile (62). Moreover, in a systemic review showed that vitamin D-fortified bread is a promising vehicle for fortification strategy effects, leading to increased serum concentrations of 25(OH)D and decreased parathyroid hormone (63). Studies show mixed attitudes toward vitamin D-fortified bread, with sensory changes impacting acceptance (64).

#### 3.3.4 Bread fortification with vitamin A

Vitamin A is crucial for vision, growth, and immune function, existing in two forms: preformed vitamin A from animal products and provitamin A carotenoids from plants. Inadequate levels can lead to night blindness, dry eyes, and weakened immunity. Vitamin A plays a vital role in cell growth, development, and maintaining healthy skin and mucous membranes. It is essential for fetal development and a strong immune system, particularly in low-and middle-income countries where deficiency is a major public health concern for young children, pregnant women, and women of reproductive age (4, 65–67). According to national survey done in Jordan at 2019, prevalence of vitamin A deficiency was 4–8% depending on serum retinol or RBP. There were no difference among household wealth, residence or sex (49).

Limited data is available on the impact of vitamin A-fortified bread in the Middle East since the countries fortifying bread with Vitamin A are only Jordan and Palestine.

#### 3.3.5 Bread fortification with B vitamins

B vitamins are essential for energy, brain function, and healthy nerves. Deficiencies in specific B vitamins, especially folate (B9) and cobalamin (B12), can lead to health issues. Pregnant women with folate deficiency have an increased risk of birth defects such as NTDs. Deficiencies in B9 and B12 can cause nerve damage, anemia, and may be linked to heart disease. A balanced diet rich in whole grains, vegetables, and animal products is recommended for adequate B vitamin intake. Fortifying bread with B vitamins can also help prevent deficiencies (6, 68, 69). A mandatory flour fortification policy with folic acid was implemented in Iran at one province in 2001 which expanded drastically to prevent the incidence of NTDs (53). A longitudinal hospital-based study coupled with two cross sectional study were conducted before (2006) and after flour fortification (2008) to assess the impact of folate fortification on NTDs and folate status in women of childbearing age involved 13,361 postpartum women longitudinally and 580 women cross-section. This study found a significant increase in folate intake after fortification, with fortified bread contributing an average of 226 μg/day. The total folate intake increased significantly from 198.3 to 413.7 μg/day. The mean Serum folate levels increased from 13.6 to 18.1 nmoL/L, folate deficiency decreased from 14.3 to 2.3%, and NTDs decreased by 31% significantly. The study concluded that mandatory flour fortification with folic acid can significantly increase serum folate levels and decrease the incidence of NTDs (53).

Initially, Fortification of bread with folate was implemented in Egypt at 2008 which was triggered by Egyptian Demographic and Health Survey (EDHS) results and showed an increases in daily folate intake of approximately 600 μg (20), however wheat flour fortification program in Egypt was ceased in 2014. Despite the challenges, Egypt’s Ministry of supply and Internal Trade (MOSIT) continues to partner with FFI to restart the program (70). A study assessing the increased folate content in Egyptian pita bread and Baladi bread found that both types were acceptable to consumers. Consumption of these fortified breads could increase average daily folate intake by 75 μg and 50 mg/100 g, respectively. However, the folate content in fortified breads was lower than the threshold level needed to decrease the incidence of NTDs. This suggests the need for further studies to assess the effectiveness of this threshold in reducing NTDs (71–73). Another study analyzed the folic acid content of wheat and soybean flour added to Baladi bread. The study found that the folic acid content in wheat flour was significantly lower than in soybean flour. After baking, the folic acid content decreased in all breads. However, the study did not explicitly state that fortifying wheat flour with folic acid is less effective than fortifying soy flour. Further studies are needed to explore the folate content and effectiveness of fortification in different flours in this region (74).

In contrast, an observational study conducted in Oman by Alasfoor et al. examined the impact of folic acid fortification in flour on birth outcomes over 15 years, from 1991 to 2006. The study analyzed the national policy that included folic acid supplementation for pregnant women in 1990 and fortified flour with folic acid in 1996. The findings of the study revealed a dramatic decline in the incidence of spina bifida, a serious neural tube defect. Prior to the fortification initiative, the rate of spina bifida ranged between 2.34 and 4.03 per 1,000 deliveries. After the implementation of the fortification, there was a sharp decline in spina bifida incidence, with rates falling from 3.06 per 1,000 deliveries in 1996 to 2.11 in 1997. By 2006, this rate had plummeted to just 0.29 per 1,000 deliveries (75). Additionally, fortifying flour with folic acid at current instance shows a decrease percentage in neural tube defects incidence per 1,000 births in KSA, Oman and Iran are (60, 70 and 31%, respectively), based on FFI report (17). This substantial decline suggests that folic acid fortification could be a valuable public health strategy for reducing birth defects when implemented with adequate fortification levels and consistent monitoring (Table 3).

**Table 3:** Overview of wheat flour fortification programs and their impact across middle eastern countries.

Country	Timeline	Coverage /compliance	Observed benefits	Reduction in deficiencies	Side effects
Egypt (20, 71–73, 75, 97)	2008: National program for wheat flour fortification. Restarted in 2019 after a pause in 2014.	95% of flour fortified by 2015.	Increased folate intake by 75 μg/day. Significant reduction in spina bifida rates from 3.06/1,000 in 1996 to 0.29/1,000 in 2006.	Neural tube defects reduced significantly.	None reported.
Iran (11, 24, 27)	2006: Mandatory wheat flour fortification.	100% of industrial flour fortified.	Serum ferritin and folate levels improved; folate deficiency dropped from 14.3 to 2.3%. NTDs reduced by 31%. Zinc-fortified bread improved absorption.	Iron deficiency rates decreased; anemia rates less responsive.	Increased oxidative stress biomarkers observed in healthy men.
Qatar (23, 26, 27)	2015: Voluntary fortification initiatives.	90% of industrial mills comply.	Data on benefits limited.	Limited data available.	None reported.
Saudi Arabia (23, 24).	2015: Voluntary fortification initiatives.	100% of industrial mills comply.	60% reduction in NTDs.	Neural tube defects significantly reduced.	None reported.
Kuwait (23, 26)	2015: Mandatory fortification.	Full compliance through one mill.	Limited data on specific outcomes.	Limited data available.	None reported.
United Arab emirates (23, 24, 26)	1996: Voluntary programs initiated.	90% of industrial flour fortified.	Improved micronutrient levels; limited long-term data.	Limited data available.	None reported.
Oman (23, 24, 29)	1996: Mandatory fortification; expanded in 2022.	89% compliance.	NTD rates reduced by 70%.	Neural tube defects significantly reduced.	None reported.
Jordan (23, 24, 26)	2006: National flour fortification program initiated.	93% coverage by 2008.	Iron deficiency anemia reduced from 28.6 to 10.7% (2019).	Iron deficiency significantly reduced.	None reported.
Iraq (23)	2000: Voluntary programs introduced.	0% industrial fortification.	No benefits observed due to lack of compliance.	No change observed due to 0% coverage.	None reported.
Palestine (26, 30)	2005: Mandatory wheat fortification.	Insufficient data on compliance.	Limited evidence of benefits.	Insufficient data available.	None reported.
Yemen (23, 26)	2001: Mandatory fortification.	100% compliance.	Limited data on outcomes.	Insufficient data available.	None reported.
Turkey (59).	Zinc-fortified bread programs implemented.	Limited implementation data available.	Improved zinc and leukocyte levels in schoolchildren with zinc deficiency.	Zinc deficiency significantly reduced in targeted populations.	None reported.
Bahrain (23, 24)	2002: Mandatory fortification	90% of the industrial flour is fortified	Limited evidence of benefits.	Limited data available.	None reported.

## 4 Discussion

### 4.1 Balancing public health and affordability

The health benefits of the Middle East’s bread fortification policies must be balanced with economic feasibility. Evaluating program costs is crucial for long-term sustainability and public health. While the economic benefits of bread fortification in reducing healthcare costs are not well-documented in the Middle East, research such as the Copenhagen Consensus’ cost–benefit analyses highlights the broader economic burden of malnutrition. Estimates suggest annual Gross Domestic Product (GDP) losses of up to 12% in poor countries (76), which can be mitigated if supplementation and fortification efforts are incorporated into existing health services and food production processes.

Supplementation programs can deliver targeted nutrients, but their reach is limited by challenges such as low awareness (77), adherence (78), and higher costs (79). In contrast, fortification leverages existing food infrastructure, reaching a broader population at a lower cost per capita. Integrating essential vitamins and minerals into a staple food like bread ensures automatic intake and improved nutritional status with minimal disruption to food chains (80). Therefore, fortification is a cost-effective strategy compared to supplementation (81).

However, a multi-pronged approach with dietary diversity and nutrition education remains crucial for long-term solutions.

### 4.2 Challenges and limitations to fortification

Bread fortification offers significant potential for addressing micronutrient deficiencies and associated health risks. However, it comes with challenges and limitations. A thorough comprehension of these challenges is crucial for devising evidence-based strategies to enhance bread fortification programs and maximize their impact on public health.

- **Inadequate coverage or compliance**: Inadequate coverage of bread fortification in the Middle East remains a pressing issue, with only 11 out of 22 countries implementing mandatory fortification programs. The coverage is inconsistent across the region, exemplified by Jordan where less than half of households have access to fortified bread on the contrary Iraq with 0%coverage. Factors contributing to this inadequacy include lack of political will, financial constraints, coordination challenges between government and private sectors, and difficulties in controlling wheat imports and small-scale flour production (82). Additionally, in some cases, manufacturers may not adhere strictly to fortification standards, reducing the effectiveness of programs.
- **Bioavailability**: Some nutrients are sensitive to processing conditions or interactions with other ingredients, impacting their stability and bioavailability. Iron poses the greatest challenge due to its impact on sensory attributes. Water-soluble iron compounds can alter color and flavor, while insoluble compounds maintain sensory properties but have limited bioavailability (83). Selecting the right iron compound for fortification is further hindered by inhibitors found naturally in food, such as phytic acid in grains or polyphenols in beverages such as tea, often consumed alongside meals (46, 84, 85). The optimal absorption of minerals can be achieved through a higher fortification level or by using iron-rich raw materials, which may offer cost-effective alternatives. Vitamin D2 from UVB-irradiated yeast has lower bioavailability than Vitamin D3 due to its interactions with the composition of yeast cell walls, particularly B-glucans, or with bile salts (86). Nevertheless, Madsen et al. revealed a reduction in the vitamin D3 content during the baking process of wheat and rye fortified bread (87). Fermentation periods surpassing 60 min, in conjunction with prolonged exposure to high temperatures, may exacerbate vitamin loss during the bread preparation process (88).
- **Socio-cultural factors**: Variations in taste, texture, color, and aroma between fortified and non-fortified bread can influence consumers’ willingness to choose fortified bread. For instance, a study showed that higher fortification with cumin and caraway seeds, aimed at enhancing fiber and nutrients, resulted in increased bitterness, color changes, and bread hardness, potentially reducing consumer acceptance (89). Encapsulation presents a promising solution to mitigate these challenges while enhancing the quality and shelf-life of bread (19, 90). To enhance consumer acceptability, education, and awareness campaigns are essential to communicate the health benefits of fortified bread and address any misconceptions or safety concerns. Before Egypt’s iron fortification program, misleading newspaper articles sparked concerns about its safety, leading to widespread misconceptions. However, proactive measures by the Health Committee, including reassuring statements on the safety of fortified foods, countered these claims, resulting in a shift toward more positive and informed media coverage (91).
- **Infrastructure Issues**: Establishing a reliable fortificant supply chain is challenging in regions with underdeveloped infrastructure or limited raw materials. The World Bank report highlight show logistical constraints hindered implementation efforts in Yemen, where infrastructure challenges limited the availability of fortified flour (92). It is also crucial to maintain the cold chain for certain sensitive fortificants, like vitamins, to preserve their potency during storage and transportation (93). These logistical complexities can result in increased costs and operational challenges for bread manufacturers and distributors, especially in resource-constrained settings. This is especially significant for an area like the Middle East which has an import-based food system mostly due to limited capacity to produce food locally.
- **Financial constraints**: The high initial costs for equipment and technology make it tough for smaller bakeries or manufacturers to participate in fortification programs. In addition, keeping these programs running requires ongoing funding and evaluation to study their impact on public health outcomes and justify continued investment. However, the cost of fortified products often renders them inaccessible to financially disadvantaged individuals. Taxes and fees on fortification equipment further exacerbate this issue (94). To address these financial challenges and ensure the sustainability of fortification initiatives, public-private partnerships and innovative financing mechanisms may provide viable solutions.
- **Political and policy barriers**: The oversight of food fortification lacks integration into legal structures, leading to inconsistent enforcement and oversight gaps. Low awareness of micronutrient deficiencies, and inadequate data to guide policy-making hinder implementation (50, 94).
- **Overconsumption of nutrients**: To guarantee the efficacy of fortification programs, ongoing monitoring of additional nutrient intake and nutritional status linked to the consumption of fortified foods is essential. Studies have highlighted the potential for iron overload in children consuming fortified bread, as evidenced by risk assessments conducted in Isfahan, Iran, which identified elevated iron intake among children (95). Additionally, unregulated fortification with folic acid has been linked to masking vitamin B12 deficiency, particularly in older adults, increasing the risk of neurological damage (96). The risks are particularly pronounced when fortified foods are consumed by non-target populations, such as children or individuals with specific health conditions. Toxicological evaluations and upper intake level assessments are essential components of effective fortification policies to prevent such adverse effects.

### 4.3 Future directions and recommendations for fortification

To enhance programs in the coming decades, the following recommendations could be considered to sustain the adoption of a multidimensional approach aimed at enhancing nutritional outcomes and addressing current challenges.

Firstly, more research is needed to evaluate fortification programs across a broader range of Middle Eastern countries, moving beyond the focus on Egypt and Iran. This will help assess the impact and effectiveness of these policies regionally while monitoring for potential micronutrient overload due to excessive fortification. Secondly, transitioning to mandatory fortification and addressing the challenges faced by small-scale mills is crucial. Strict quality standards must be maintained to ensure that fortified bread meets established nutritional values, which can enhance consumer confidence and acceptance. Exploring innovative fortification methods can also help manufacturers overcome technical obstacles. Thirdly, fortification policies should extend beyond established nutrients like iron and vitamin D to include essential micronutrients, such as vitamin A, to address specific nutritional gaps. Tailored fortification programs are vital for meeting the needs of different populations, especially at-risk groups. Finally, combining fortification with nutrition education can inform consumers about the benefits and safety of fortified bread. Strengthening the supply chain will also mitigate challenges in procuring and storing ingredients. Overall, establishing common standards and guidelines will promote consistency and quality, supporting cross-border trade. Capacity building for bread factories, particularly smaller facilities, is essential to enhance technical capabilities and achieve better nutritional outcomes in communities.

## 5 Conclusion

In conclusion, many studies on the effectiveness of fortification programs of bread in the Middle East are mostly centered on countries such as Egypt and Iran. This indicates a gap in research coverage for other countries in the region, highlighting the need for more studies to evaluate fortification programs of bread in a wider range of Middle Eastern countries. This would provide a more comprehensive understanding of the region’s impact and effectiveness of fortification policies.

Countries like Egypt, Iran, Saudi Arabia, Jordan and Oman have successfully reduced micronutrient deficiencies, particularly neural tube defects (NTDs) and iron deficiency anemia, through fortification programs. For instance, Egypt’s initiatives have lowered NTD rates, while Iran has seen improvements in folate and ferritin levels and reduced iron deficiency rates (96). However, challenges such as inadequate coverage, compliance issues, and lack of long-term data are present in some regions, including Qatar and Palestine, where limited data hinders the assessment of fortification benefits. Countries like Iraq have not seen significant improvements due to poor compliance, with 0% industrial fortification.

Overall, the evidence suggests that flour fortification is effective in addressing micronutrient deficiencies across the region without micronutrient overload, though the success of these programs heavily depends on full compliance, coverage, and sustained monitoring. There is also a need for more comprehensive data and evaluation to further assess the long-term impact and potential side effects, such as increased oxidative stress observed in some studies, and ensure the safety and effectiveness of these interventions.

## Footnotes

^1^https://static1.squarespace.com/static/5e1df234eef02705f5446453/t/5f7cb553a0fbb52e16e47a80/1602008406644/FolicAcidBackground.pdf.
